# Modern Management of the Axilla in HER2-Negative Hormone Receptor-Positive Early Breast Cancer Upfront Surgery: Toward De-Escalation and Individualization

**DOI:** 10.3390/cancers18010131

**Published:** 2025-12-30

**Authors:** Halima Abahssain, David Pasquier, Khaoula Laabid, Meryem Barani, Sébastien Borges, Stephen Poitureau, Ghizlane Bettache, Thi-Lan-Anh Nguyen, Mbolam Bytha, Joseph Rodriguez, Antoine Lemaire, Giuseppe Curigliano, Amine Souadka

**Affiliations:** 1 Oncology and Medical Specialties Department, Valenciennes General Hospital, 59300 Valenciennes, France; 2 Equipe de Recherche en Oncologie Translationnelle (EROT), Faculty of Medicine and Pharmacy, University Mohammed V in Rabat, Rabat 10100, Morocco; 3 Academic Department of Radiation Oncology, Centre Oscar Lambret, 59000 Lille, France; 4 CNRS, Centrale Lille, UMR 9189–CRIStAL, University of Lille, 59000 Lille, France; 5 Radiation Oncology Department, Mohammed VI University Hospital, Marrakesh 40000, Morocco; 6 Supportive Care Department, Valenciennes General Hospital, 59300 Valenciennes, France; 7 Department of Oncology and Hemato-Oncology, University of Milan, 20133 Milan, Italy; 8 Surgical Oncology Department, National Institute of Oncology, University Mohammed V in Rabat, Rabat 10100, Morocco

**Keywords:** HER2-negative, hormone receptor-positive, breast cancer, sentinel lymph node biopsy, radiotherapy, CDK4/6 inhibitors

## Abstract

Axillary management in early-stage, HER2-negative, hormone receptor-positive breast cancer has undergone major changes in recent years. While axillary lymph node dissection (ALND) was once considered essential for staging and regional control, increasing evidence supports the safety of surgical de-escalation in selected patients. At the same time, systemic therapies such as CDK4/6 and PARP inhibitors rely on nodal burden to define eligibility, raising new challenges in balancing oncologic benefit with treatment-related morbidity. This narrative review summarizes current strategies in axillary management for patients undergoing upfront surgery for HR-positive, HER2-negative early breast cancer. It explores the role of sentinel lymph node biopsy (SLNB), the indications for ALND, the integration of adjuvant systemic therapy, and the emerging role of radiotherapy and predictive tools in guiding individualized treatment decisions. Key randomized trials including Z0011, AMAROS, SENOMAC, SOUND, and INSEMA have demonstrated that omission of ALND is safe in patients with limited nodal involvement, especially when combined with whole-breast or regional nodal radiotherapy. However, trials such as MonarchE and OlympiA have introduced systemic therapies whose indications are closely tied to nodal status, prompting reconsideration of the extent of axillary staging. Advances in imaging and risk stratification tools offer new avenues for safely limiting surgical intervention while preserving access to systemic options. In conclusion, modern axillary management in HR-positive, HER2-negative breast cancer involves navigating the intersection between de-escalated surgery and risk-adapted systemic therapy. Future strategies should prioritize individualized care, incorporating tumor biology, imaging findings, and patient preferences, with multidisciplinary collaboration playing a central role in optimizing outcomes.

## Simple Summary

Axillary management in HER2-negative, hormone receptor-positive early breast cancer has shifted toward surgical de-escalation, supported by randomized trials demonstrating that completion ALND can be safely omitted in selected patients with limited nodal involvement, particularly when radiotherapy or genomic risk profiling is integrated. Systemic treatment strategies increasingly incorporate nodal burden and biologic profil to guide adjuvant therapy decisions, while predictive tools and high-resolution axillary ultrasound help estimate additional nodal involvement without routine dissection. At the same time, the therapeutic benefit of CDK4/6 and PARP inhibitors must be balanced against substantial risks of severe arm morbidity when ALND is used solely to meet drug-eligibility thresholds. A multidisciplinary, risk-adapted approach that integrates tumor biology, imaging, predictive modeling, minimal residual disease assessment, and patient preferences is now central to delivering individualized care while minimizing morbidity and preserving oncologic safety.

## 1. Introduction

Axillary management remains central to staging and treatment in early breast cancer. Historically, axillary lymph node dissection (ALND) was considered standard, based on the belief that complete nodal clearance would improve outcomes. However, its survival benefit was never clearly demonstrated. In contrast, its complications including lymphedema, sensory neuropathy, and restricted shoulder mobility are well documented.

Over time, breast cancer prognosis has evolved to consider both tumor biology and clinical factors such as tumor size and nodal status. HER2-negative, hormone receptor-positive (HR+) breast cancer is the most prevalent subtype. For these patients, endocrine therapy remains the cornerstone of systemic treatment. Nonetheless, nodal involvement continues to guide prognosis and therapeutic decisions. Pan et al. reported a 10-year risk of distant recurrence rising from 13% in node-negative patients to 20% with one to three positive nodes and 34% with four to nine [1].

The introduction of CDK4/6 and PARP inhibitors has expanded adjuvant options for high-risk patients. However, eligibility for these therapies often depends on nodal burden, reinforcing the importance of accurate axillary staging, even as surgical approaches become less invasive.

The shift towards sentinel lymph node biopsy (SLNB) has significantly reduced surgical morbidity. Yet, this de-escalation presents a clinical paradox: while fewer nodes are removed surgically, systemic treatment decisions increasingly depend on nodal count. This has led to a growing need for individualized management strategies that balance oncologic safety with quality of life.

This review examines current approaches to axillary management in HER2-negative, hormone receptor-positive early breast cancer, with a focus on surgical de-escalation, systemic therapy integration, radiotherapy alternatives, and emerging tools for personalized decision-making.

## 2. Methodology

This narrative review summarizes current strategies for axillary management in patients with HER2-negative, hormone receptor-positive early breast cancer undergoing upfront surgery. The focus is on surgical de-escalation, integration of systemic therapies, radiotherapy alternatives, and predictive tools guiding individualized care.

Relevant studies were identified through PubMed, MEDLINE, and Embase searches using keywords such as “sentinel lymph node biopsy,” “axillary lymph node dissection,” “CDK4/6 inhibitors,” “PARP inhibitors,” “axillary radiotherapy,” and “de-escalation.” Only peer-reviewed publications were included, encompassing randomized controlled trials, cohort studies, systematic reviews, and guidelines from ASCO, NCCN, and ESMO.

Priority was given to studies influencing current practice or addressing controversies in axillary management. Trials evaluating omission of ALND or SLNB, systemic therapy eligibility, and predictive modeling for nodal burden were emphasized.

Key trials analyzed include ACOSOG Z0011, AMAROS, SENOMAC, SOUND, INSEMA, MonarchE, RxPONDER, and NATALEE. Each study was appraised for methodological quality, endpoints (disease-free survival, overall survival, quality of life), and clinical relevance.

## 3. Historical Evolution and De-Escalation of Axillary Surgery

### 3.1. From ALND to Sentinel Node Biopsy

Axillary lymph node dissection (ALND) was long considered the standard for early breast cancer, based on the belief that complete nodal clearance would improve survival. This rationale extended, in some cases, to internal mammary node dissection [2,3].

However, ALND’s survival benefit was never definitively proven, while its complications lymphedema, neuropathy, and restricted shoulder mobility became well recognized [4,5].

De-escalation efforts began in the 1970s. Fisher et al. showed that among cN0 patients, 10-year disease-free survival (DFS) and overall survival (OS) were similar whether treated with radical mastectomy, total mastectomy with nodal irradiation, or delayed ALND only if nodes were positive [3].

As the high rate of negative ALNDs and its morbidity became apparent, attention shifted to less invasive approaches. Sentinel lymph node biopsy (SLNB), introduced by Veronesi et al., allowed reliable axillary staging while sparing node-negative patients from ALND [6].

Large trials, including NSABP B-32, confirmed that SLNB provided comparable disease control to ALND in cN0 patients, with significantly fewer complications [7,8].

### 3.2. Omission of ALND After Positive Sentinel Nodes

The next step in de-escalation addressed the need for ALND in patients with limited sentinel node involvement. The ACOSOG Z0011 trial demonstrated that in cN0 patients with T1–T2 tumors and one or two positive sentinel nodes, ALND could be safely omitted when breast-conserving surgery and radiotherapy were performed [9].

The SENOMAC trial reinforced these findings, confirming that SLNB alone was non-inferior to completion ALND in cN0 patients with T1–T3 tumors and up to two macrometastatic sentinel nodes [10].

Similar conclusions were drawn from AMAROS, IBCSG 23-01, AATRM, OTOASOR, SINODAR-ONE, and the ongoing POSNOC trial, supporting omission of ALND in low-volume nodal disease (see Table 1).

### 3.3. Beyond SLNB: Extreme De-Escalation

Although SLNB significantly reduces morbidity compared to ALND, it is not without risk. Lymphedema rates of ~6% and other morbidities up to 10% have been reported [17,18]. This has prompted studies evaluating omission of axillary surgery altogether in select patients.

The SOUND trial enrolled patients with tumors ≤ 2 cm and negative axillary ultrasound, randomizing them to SLNB or observation. Distant DFS was equivalent in both groups [19]. However, caution is warranted in younger patients and high-grade tumors, where nodal evaluation may still influence treatment decisions.

The INSEMA trial, a large randomized study comparing SLNB to no axillary surgery in cN0 patients undergoing breast-conserving surgery, also found no significant difference in outcomes after six years of follow-up [20] (see Table 2).

Taken together, these findings suggest that, in properly selected patients, axillary surgery may no longer be necessary for staging or treatment. However, patient selection remains critical, and the approach must be individualized based on clinical and biological risk.

## 4. When Is ALND Still Relevant?

While ALND is no longer routinely required in early breast cancer, it remains indicated in select scenarios.

ALND continues to be appropriate for patients with confirmed axillary involvement (cN+) who undergo upfront surgery rather than neoadjuvant therapy. In these cases, assessing axillary burden is essential, as eligibility for CDK4/6 or PARP inhibitors depends on nodal count. In the MonarchE trial, patients with ≥4 involved nodes, or 1–3 nodes plus high-risk features, benefited from adjuvant abemaciclib [25]. Thus, ALND remains recommended regardless of planned systemic therapy.

The introduction of CDK4/6 inhibitors in the adjuvant setting has raised new questions about ALND’s role. MonarchE defined high-risk HR-positive, HER2-negative disease as ≥4 positive nodes, or 1–3 positive nodes with high-risk features such as tumor size ≥ 5 cm or grade 3 histology. These criteria have led some clinicians to consider ALND in patients with 1–2 positive sentinel nodes to uncover additional metastases and qualify for abemaciclib.

This approach, however, remains controversial. ALND was not mandated in MonarchE, and the proportion of patients who underwent it was not reported [26]. Similarly, in the RxPONDER trial where patients with 1–3 positive nodes and a recurrence score < 26 were randomized to chemotherapy or not ALND was not required. In postmenopausal women, no benefit from chemotherapy was observed, reinforcing the value of gene expression assays in guiding treatment when nodal involvement is limited [27].

These studies have shifted ALND’s rationale from local control to systemic therapy eligibility. Still, neither MonarchE nor RxPONDER was designed to assess surgical strategy, and their findings must be interpreted cautiously in the broader context of de-escalation [28].

Balancing potential systemic benefit against ALND-associated morbidity remains complex. In a post hoc SENOMAC analysis of 1342 patients completing quality-of-life questionnaires, severe or very severe arm function impairment occurred in 13% of ALND patients versus 4% after SLNB alone (*p* < 0.0001) [29].

Quantitative modeling from the SENOMAC trial further underscores this imbalance when axillary lymph node dissection is performed solely to identify patients eligible for adjuvant CDK4/6 inhibitors. Post hoc analyses estimated that approximately 104 completion ALNDs would be required to prevent a single invasive disease-free survival event associated with abemaciclib, while nine patients would experience severe or very severe arm morbidity. This unfavorable trade-off strongly argues against routine ALND in patients with limited sentinel node involvement when the sole purpose is to meet systemic therapy eligibility thresholds.

ALND remains essential in inflammatory breast cancer (IBC), a biologically aggressive subtype with poor prognosis despite multimodal therapy. In IBC, ALND is typically performed after neoadjuvant treatment, especially when total mastectomy is indicated [30,31]. A population-based study using the U.S. National Cancer Database found that in cN2–N3 IBC, overall survival was significantly higher when ≥10 lymph nodes were resected, regardless of final pathology [32].

For patients with locally advanced disease responding well to neoadjuvant chemotherapy (NACT), the use of SLNB remains under investigation. While feasible in selected cases, its application after downstaging from bulky axillary disease is controversial. A meta-analysis of 17 studies showed high false-negative and low identification rates for SLNB in patients downstaged to ycN0 after NACT [33]. Consequently, ALND remains preferred in patients initially presenting with cN2 or cN3 disease, even with clinical response.

The SENOMAC post hoc analysis also explored ALND’s utility in identifying patients with ≥4 positive nodes who may benefit from abemaciclib. However, the associated morbidity was substantial, and authors concluded that routine ALND in this context likely causes more harm than benefit [29,33].

The role of ALND in patients undergoing mastectomy is also debated. In SENOMAC, about one-third of patients underwent mastectomy and achieved favorable outcomes without ALND when treated with adjuvant radiotherapy. Similarly, a sub-analysis of the SINODAR-ONE trial showed no significant difference in five-year recurrence or survival between ALND and SLNB groups in patients managed with mastectomy. The ongoing POSNOC trial is expected to clarify this further [15].

## 5. Integration of Systemic Therapy

### 5.1. Endocrine Therapy

Hormone receptor-positive, HER2-negative breast cancer is the most common molecular subtype, accounting for approximately 70–75% of early-stage cases [34,35]. For these patients, endocrine therapy is the foundation of systemic treatment, with tamoxifen or aromatase inhibitors used based on menopausal status.

HR-positive disease shows a distinct temporal recurrence pattern. Saphner et al. demonstrated that, unlike triple-negative or HER2-positive tumors, HR-positive cancers are associated with a sustained risk of recurrence beyond five years, often referred to as a “carryover effect.” [36].

Nodal involvement further amplifies this risk, and long-term follow-up confirms that patients Nodal involvement further amplifies recurrence risk. Long-term data confirm that patients with ≥4 positive nodes face significantly higher rates of distant relapse. Strategies to reduce recurrence in high-risk patients include extended endocrine therapy, ovarian suppression in premenopausal women, and adjuvant chemotherapy guided by clinical and genomic factors.

### 5.2. Endocrine Resistance and Molecular Biomarkers

Resistance to endocrine therapy in hormone receptor-positive, HER2-negative breast cancer represents a major determinant of long-term outcomes [37]. Acquired resistance is frequently driven by molecular alterations that are not captured by traditional clinicopathologic variables, including axillary nodal burden. Among these, ESR1 ligand-binding domain mutations are well recognized mechanisms of resistance to endocrine therapy, increasingly detected through circulating tumor DNA (ctDNA), leading to constitutive estrogen receptor activation and impacted endocrine sensitivity [38,39].

In parallel, aberrations in the PI3K/AKT/mTOR signaling pathway, particularly activating PIK3CA mutations, contribute to endocrine resistance and tumor proliferation [40,41]. These alterations have clear therapeutic implications, as demonstrated by the efficacy of PI3K and mTOR inhibitors in advanced disease [42,43]. Although not yet routinely incorporated into early-stage treatment algorithms, these molecular markers are likely to assume an increasingly important role in refining adjuvant therapy selection.

Importantly, these biological drivers highlight the limitations of relying on axillary nodal count alone to inform systemic therapy decisions. As breast cancer management increasingly incorporates biomarker-driven and molecularly targeted treatments, future risk-stratification frameworks are likely to integrate genomic alterations, validated molecular biomarkers, and measures of minimal residual disease in addition to conventional anatomical staging. Such an approach may reduce the need for axillary lymph node dissection performed solely to meet systemic treatment eligibility criteria, thereby supporting surgical de-escalation while maintaining appropriate oncologic decision-making.

### 5.3. Chemotherapy Fundamentals

In the adjuvant setting, chemotherapy decisions are based on tumor size, grade, lymphovascular invasion, Ki-67, menopausal status, and nodal status. Multigene expression assays such as Oncotype DX, MammaPrint, and EndoPredict have become integral tools in refining adjuvant systemic therapy decisions in HR-positive, HER2-negative early breast cancer [44,45,46]. These assays provide prognostic and predictive information that is independent of, and often complementary to, axillary nodal status. Data from trials such as RxPONDER have demonstrated that genomic risk can supersede nodal burden in guiding chemotherapy decisions, particularly in postmenopausal women with limited nodal involvement. This study found no benefit from adjuvant chemotherapy in postmenopausal women with 1–3 positive nodes and a recurrence score below 26. In contrast, premenopausal women in the same group experienced improved outcomes, suggesting a possible endocrine effect of chemotherapy [47].

In this context, multigene signatures challenge the historical primacy of extensive axillary staging for treatment stratification. Their increasing use supports a paradigm in which escalation or de-escalation of systemic therapy is driven primarily by tumor biology rather than surgical extent alone. However, despite these tools, up to 20% of patients with hormone receptor-positive, HER2-negative disease experience disease recurrence within 10 years, underscoring the need for additional strategies beyond endocrine therapy and chemotherapy [48]. This has led to growing interest in adjuvant use of targeted agents, particularly CDK4/6 and PARP inhibitors.

### 5.4. CDK4/6 Inhibitors

Cyclin-dependent kinase (CDK) 4 and 6 inhibitors have transformed the treatment landscape of HR-positive, HER2-negative breast cancer. Abemaciclib, ribociclib, and palbociclib are standard in metastatic setting, where they improve progression-free survival. Ribociclib has also shown overall survival benefits in multiple phase III trials [49,50].

These successes prompted trials of CDK4/6 inhibitors in early-stage disease. The MonarchE trial assessed abemaciclib plus endocrine therapy in patients with high-risk features, either ≥4 positive nodes, or 1–3 nodes with tumor size ≥ 5 cm or grade 3 histology. Abemaciclib significantly improved invasive disease-free survival (HR 0.75) and reduced distant recurrence (HR 0.72) [51]. After a median follow-up of 76.2 months, abemaciclib in association with ET resulted in a 15.8% lower risk of death than ET (HR 0.842, *p* = 0.027) leading to its approval in the adjuvant setting for selected patients [52].

However, abemaciclib eligibility, tied to nodal burden, has complicated axillary management. For patients with 1–2 positive sentinel nodes, clinicians may consider completion ALND to identify ≥4 involved nodes. This conflicts with prior evidence from Z0011 and AMAROS supporting omission of ALND in low-burden disease.

The issue is compounded by the lack of surgical details in MonarchE. ALND was not mandated, and the number of patients undergoing completion dissection was not reported, making it unclear how nodal staging affected outcomes.

The NATALEE trial evaluated ribociclib in a broader population, including node-negative patients with high-risk features such as T2 grade 2 tumors with high Ki-67, or T3–T4 tumors of any grade [53]. Ribociclib continued to demonstrate iDFS benefit over NSAI alone (HR 0.72) after a median follow up of 44.2 months [54]. These expanded criteria offer greater flexibility and reduce the need for extensive axillary surgery to reach nodal thresholds.

Together, these trials highlight the tension between therapeutic escalation and surgical de-escalation. CDK4/6 inhibitors offer real benefit, but their integration into early-stage management calls for nuanced decision-making. Predictive tools and individualized assessments may help guide therapy without reversing progress in minimizing axillary morbidity (see Table 3).

### 5.5. PARP Inhibitors (Poly (Adenosine Diphosphate-Ribose) Polymerase Inhibitor)

PARP inhibitors represent a targeted approach for patients with germline BRCA mutations. These agents exploit synthetic lethality by blocking DNA repair, leading to cell death in tumors deficient in homologous recombination repair (HRR) pathways [55,56]. This strategy is particularly effective in BRCA1/2-mutated, HR-positive, HER2-negative breast cancer.

In metastatic disease, olaparib and talazoparib have shown clinical efficacy in germline BRCA-mutated, HER2-negative patients. The OlympiAD and EMBRACA trials demonstrated significantly improved progression-free survival over standard chemotherapy, leading to regulatory approval [57,58].

The benefit of PARP inhibition has since been demonstrated in early-stage disease. The OlympiA trial evaluated one year of olaparib in high-risk, HER2-negative breast cancer with confirmed BRCA1/2 mutations. Eligibility required either triple-negative disease with residual tumor post-neoadjuvant chemotherapy or hormone receptor-positive tumors with a CPS + EG score > 3 after neoadjuvant therapy, or ≥4 positive lymph nodes after upfront surgery [59].

Approximately 18% of OlympiA participants had hormone receptor-positive tumors. In this subgroup, olaparib significantly improved invasive disease-free and overall survival, independent of receptor status. However, patients receiving upfront surgery had to present with ≥4 involved lymph nodes to be eligible again linking access to systemic therapy with axillary burden [59].

This requirement raises important questions about axillary staging in BRCA-mutated patients otherwise suitable for surgical de-escalation. While ALND may uncover patients eligible for olaparib, it also increases morbidity.

Emerging strategies like tailored axillary surgery (TAS) after neoadjuvant therapy may offer a compromise enabling accurate staging while avoiding the complications of full dissection. As systemic options expand, aligning axillary staging with therapeutic thresholds will be crucial to improving outcomes in BRCA-mutated, HR-positive, HER2-negative early breast cancer.

## 6. Radiotherapy as an Alternative to Surgery

### 6.1. Evidence Supporting Axillary Radiotherapy

Radiotherapy (RT) plays a critical role in reducing local recurrence in early breast cancer and has become central to axillary management as surgery becomes more conservative. Several studies have shown that appropriately targeted RT can provide regional control comparable to axillary lymph node dissection (ALND), with fewer complications.

Meta-analyses from the Early Breast Cancer Trialists’ Collaborative Group (EBCTCG) demonstrated that adjuvant RT significantly reduces breast cancer mortality following both breast-conserving surgery and mastectomy in node-positive disease [60,61].

Advances in radiation planning now allow for improved dose conformity and reduced exposure to adjacent organs, minimizing toxicity [62].

The ACOSOG Z0011 and AMAROS trials were pivotal in reshaping axillary management. In Z0011, patients with one or two positive sentinel nodes who received whole-breast irradiation without targeted nodal RT or ALND had low regional recurrence [9]. Retrospective data suggest that incidental coverage of axillary levels I and II with high tangents may have contributed to this outcome [63,64].

The AMAROS trial directly compared axillary RT to ALND in patients with positive sentinel nodes and clinically negative axilla. Both approaches achieved similar regional control, but RT caused significantly less lymphedema (11.9% vs. 24.5%, *p* < 0.001) [11]. These results validated axillary RT as a suitable alternative in selected patients.

In this context, early involvement of radiation oncologists is important to optimize regional control while limiting treatment-related morbidity. Multidisciplinary discussion prior to surgical decision-making is particularly relevant in situations such as mastectomy, limited sentinel node involvement, or uncertainty regarding the need for regional nodal irradiation. Early integration of radiotherapy expertise may help avoid unnecessary axillary surgery while maintaining oncologic safety.

### 6.2. Clinical Integration and Ongoing Trials

Current guidelines recommend regional nodal irradiation (RNI) when ALND is omitted, with field extent individualized based on patient risk. Clinical algorithms and nomograms now guide whether supraclavicular or internal mammary fields should be included [65,66]. However, in the era of modern systemic therapy and surgical de-escalation, the optimal extent of RNI remains under study.

Ongoing trials such as TAILOR-RT and T-REX aim to refine RT recommendations in biologically low-risk, node-positive breast cancer. These trials focus on ER-positive, HER2-negative patients to evaluate whether RNI can be safely omitted without compromising outcomes [67].

Until their results are available, decisions should consider nodal burden, tumor biology, and comorbidities. For selected patients, RT offers a non-surgical option for regional control aligned with current goals of reducing treatment-related morbidity while maintaining oncologic safety.

## 7. Emerging Technologies and Risk Profiling

### 7.1. Advances in Axillary Imaging

Preoperative assessment of the axilla is essential for accurate staging and treatment planning in early breast cancer. With the increasing emphasis on surgical de-escalation, imaging plays a key role in identifying patients who may safely avoid axillary lymph node dissection (ALND).

High-resolution axillary ultrasound remains the primary imaging modality used to evaluate nodal status prior to surgery or neoadjuvant therapy. When findings are suspicious, fine needle aspiration (FNA) or core needle biopsy is often performed to confirm metastatic involvement. However, the predictive value of ultrasound can vary significantly depending on the operator’s expertise and the criteria used to define abnormal lymph nodes [68,69].

Recent efforts aim to refine ultrasound interpretation. Loonis et al. reported that features like cortical thickness > 4 mm, fatty hilum loss, and diffuse irregularity correlate with metastasis. In contrast, patients with cortical thickness < 4 mm may safely proceed to sentinel lymph node biopsy (SLNB) without FNA [70].

Comparative studies show that while MRI has the highest sensitivity (83%) and PET/CT the highest specificity (94%), ultrasound remains the most practical in routine care [68,70]. The SOUND trial used ultrasound to select patients for SLNB omission; patients with tumors ≤ 2 cm and negative ultrasound were randomized to SLNB or observation, with FNA used when nodes appeared suspicious [19].

Several retrospective analyses have shown that the combined use of ultrasound and MRI can yield similar diagnostic accuracy, suggesting that dual imaging may not be necessary for routine staging [71]. Still, multimodal imaging can be helpful in borderline cases.

Overall, axillary imaging serves as a gatekeeper for surgical de-escalation, with axillary ultrasound currently representing the preferred and most practical tool for axillary assessment [25]. As technology and image interpretation continue to improve, this approach may eventually allow selected low-risk patients to avoid sentinel lymph node biopsy altogether.

### 7.2. Predictive Tools for Node Burden

Because eligibility for adjuvant therapies like CDK4/6 and PARP inhibitors depends on nodal burden, accurately estimating lymph node involvement without ALND has become increasingly relevant. Multiple predictive models have been developed to estimate the likelihood of ≥4 positive nodes in patients with limited sentinel node involvement.

One of the earliest, the MSKCC nomogram, incorporates tumor size, histology, grade, lymphovascular invasion, and receptor status to predict non-sentinel node involvement [72]. These models are now available as clinical decision tools.

More recent efforts target prediction of ≥4 positive nodes a critical threshold in MonarchE eligibility. Yang et al. developed a nomogram using tumor size, abnormal ultrasound, calcifications, and number of positive sentinel nodes. In their study, 17.3% of patients with 1–2 positive sentinel nodes had ≥4 involved nodes on ALND [73].

Model performance depends on input quality and population applicability. Many rely on postoperative features, limiting preoperative utility. Prospective validation is needed before routine use.

Some investigators have explored intraoperative findings and biologic markers. Houvenaeghel et al. compared pre- and postoperative models and found higher accuracy with postoperative data, though it delays decisions [74].

In clinical practice, predictive tools, when integrated with axillary ultrasound, may aid risk stratification, promote surgical de-escalation while balance oncologic safety with treatment-related morbidity as systemic therapy indications expand.

## 8. Controversies and Future Directions

### 8.1. Reconsidering ALND to Access CDK4/6 Inhibitors

The integration of CDK4/6 inhibitors into early breast cancer care has raised questions about the extent of axillary staging needed for eligibility. Trials like MonarchE and NATALEE used nodal burden specifically ≥4 positive nodes as a key criterion, prompting consideration of axillary lymph node dissection (ALND) solely to identify patients eligible for systemic escalation.

While abemaciclib has reduced invasive disease-free events in high-risk patients, its broader utility is debated. Tannock et al. argue that without a demonstrated overall survival benefit, prolonged CDK4/6 therapy two years for abemaciclib and three for ribociclib may not be warranted in all patients, especially given the toxicity profile [75]. In MonarchE, over 60% of patients required dose reductions, and discontinuation rates were high. The absolute benefit preventing approximately five events per 100 patients must be balanced against treatment burden and potential morbidity from extended axillary surgery.

Methodological differences across trials complicate interpretation. Divergent censoring strategies between positive (MonarchE, NATALEE) and negative (PALLAS) trials may partly explain inconsistent outcomes [75,76,77].

Biomarkers such as circulating tumor DNA (ctDNA) may refine treatment decisions. In MonarchE, ctDNA was found in 87% of patients who recurred, versus 15% who remained recurrence-free [78]. These findings suggest ctDNA could better predict benefit from CDK4/6 inhibition than nodal count alone.

Given these concerns, ALND performed solely to meet drug eligibility criteria warrants reconsideration. The risks of surgical and systemic overtreatment must be weighed against personalized estimates of recurrence risk.

### 8.2. Broadening Systemic Therapy Criteria and the Role of Multidisciplinary Teams

The NATALEE trial offered a more flexible framework by allowing ribociclib eligibility to be defined by tumor characteristics including size, grade, Ki-67, and genomic risk in addition to nodal status. This approach may reduce reliance on axillary lymph node dissection (ALND) for systemic therapy decision-making.

**Figure 1:**
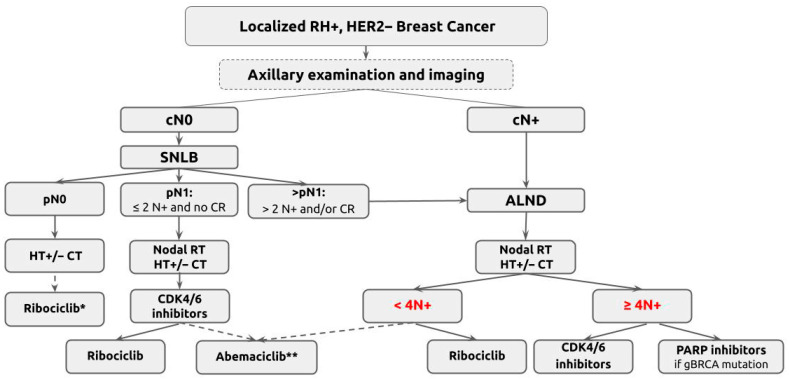
Actual algorithm for axillary management of upfront surgery for HER2-negative HR-positive early breast cancer. * For T3 or T4. For T2 if grade III or Grade II and Ki67 ≥ 20% or GII and RS > 25. ** For T ≥ 5 cm or Grade III. HR: Hormone receptors. cN0: clinically node-negative. cN+: clinically node-positive. SLNB: Sentinel lymph node biopsy. ALND: Axillary lymph node dissection. N: Node. CR: Capsular rupture. RT: Radiation therapy. HT: Hormone therapy.

As eligibility criteria broaden and axillary decision-making becomes more complex at the intersection of surgical de-escalation, radiotherapy, and systemic therapy escalation, coordinated multidisciplinary care is increasingly essential. Early and integrated input from breast surgeons, medical and radiation oncologists, radiologists, pathologists, and molecular specialists is critical to balance oncologic benefit against treatment-related morbidity, particularly in borderline situations involving nodal thresholds, eligibility for targeted therapies, or the choice between axillary surgery and radiotherapy (Figure 1).

In this evolving landscape, the roles of surgery and radiotherapy must be continually reassessed within a risk-adapted framework that integrates imaging, histopathology, genomic profiling, and patient preferences. Multidisciplinary tumor boards remain central to delivering individualized, evidence-based axillary management aimed at optimizing outcomes while minimizing harm.

## 9. Conclusions

The management of the axilla in hormone receptor-positive, HER2-negative early breast cancer has undergone a paradigm shift, moving steadily toward de-escalation. Sentinel lymph node biopsy (SLNB) has replaced axillary lymph node dissection (ALND) in many scenarios, supported by evidence demonstrating equivalent oncologic outcomes with significantly reduced morbidity.

At the same time, the increasing use of adjuvant systemic therapies particularly CDK4/6 and PARP inhibitors has reintroduced the importance of nodal burden in treatment selection. This dual dynamic creates a clinical paradox: as surgical management becomes less invasive, systemic therapy decisions may still hinge on data traditionally obtained through more extensive surgery.

To navigate this complexity, a multidisciplinary, risk-adapted approach is essential. Advances in imaging, predictive modeling, and molecular profiling offer promising avenues to individualize care without defaulting to overtreatment. Emerging biomarkers, such as circulating tumor DNA, may further refine patient selection for therapy escalation, helping to avoid unnecessary ALND and its associated morbidity.

Ultimately, the goal remains to achieve optimal oncologic control while minimizing harm. Future trials should focus on validating non-invasive strategies for axillary assessment and redefining therapeutic eligibility criteria to better align with evolving surgical practice. Until then, decisions should be guided by a careful balance of tumor biology, patient preference, and the best available evidence.

## Figures and Tables

**Table 1:** Key trials supporting de-escalation in axillary management by omitting ALND.

Trial Name	Population	Intervention	Key Findings	Reference
ACOSOG Z0011	Clinically node-negative (cN0).	SLNB vs. SLNB + ALND.	No difference in survival or recurrence for 1–2 positive SLNs; omission of ALND is safe.	[9]
AMAROS	cT1–2 with SLN+.	ART vs. ALND.	ART non-inferior to ALND in terms of local control, with less morbidity.	[11]
IBCSG 23-01	cT2 cN0, and 1 or more micrometastatic SLNs with no extracapsular extension.	SLNB vs. ALND.	SLNB non-inferior to ALND.	[12]
AATRM 048/13	T < 3.5 cm, cN0, micrometastatic SLN.	ALND or clinical follow-up.	No differences in DFS and OS.	[13]
OTOASOR	cN0 and cT ≤ 3 cm. SLNB.	ALND or axillary nodal irradiation	- No significant difference in axillary recurrence. - No significant difference in terms of OS.	[14]
POSNOC	cT1–T2, unifocal or multifocal, and 1 or 2 macrometastases at SLNB, with or without extranodal extension.	adjuvant therapy alone, in the standard care group they receive ANC or axillary RT. Surgical axillary node clearance (ANC) or radiotherapy to the axilla (ART).	Ongoing.	[15]
SENOMAC	cN0, cT1–T3, and one or two SLN macrometastases.	ALND vs. SLNB.	- non-inferiority; - ALND carries a substantial risk of severe or very severe arm morbidity.	[10]
SINODAR ONE	cT1–2 and one or two macrometastatic SLN.	removal of ≥10 axillary level I/II non-SLNs followed by adjuvant therapy or no further axillary treatment.	SLNB only, not inferior to ALND.	[16]

**Table 2:** Key trials supporting de-escalation in axillary management by omitting SLNB.

Trial Name	Population	Intervention	Key Findings	Reference
CALGB 9343	70 years or older with stage I.	Lumpectomy; received tamoxifen plus radiation therapy or tamoxifen alone.	No significant differences in time to mastectomy, time to distant metastasis, breast cancer-specific survival, or OS between the two groups.	[21]
INSEMA	cN0, T1 or T2 (tumor size, ≤5 cm), breast-conserving surgery.	Omission of axillary surgery vs. sentinel lymph node biopsy.	Omission of surgical axillary staging was non-inferior to sentinel lymph node biopsy.	[20]
BOOG 2013-08	Clinically node-negative T1–2 invasive breast cancer, conserving therapy.	Sentinel lymph node biopsy versus no sentinel lymph node biopsy.	Non-inferior regional control, distant-disease-free survival, and overall survival.	[22]
SOAPET study	T < 5 cm, planned BCS + whole breast radiation.	SLNB vs. observation.	Stage 1: NPV at 6 months Stage 2: DFS and LRFS at 5 years.	[23]
SOUND	BC up to 2 cm and a negative preoperative axillary ultrasonography.	Omission of axillary surgery vs. SLNB.	Omission of axillary surgery was noninferior to SLNB.	[19]
NAUTILUS study	Clinical stage T1–2 and cN0 breast cancer patients receiving breast-conserving surgery. Axillary ultrasound is mandatory before surgery.	No-SLNB (test) and SLNB (control) groups.	Ongoing. The trial will provide the oncological safety of the omission of SLNB in patients undergoing breast-conserving surgery and receiving whole-breast radiation.	[24]

**Table 3:** Trials supporting using CDK4/6 inhibitors in adjuvant settings.

Trial	Population	Intervention	Key Findings	Reference
MonarchE	≥4 ALNs or 1–3 ALNs + high-risk features.	Endocrine therapy ± abemaciclib.	Improved iDFS and DRFS; HR~0.75. Improved OS; HR 0.842, *p* = 0.027.	[51,52]
NATALEE	Node-positive or high-risk node-negative (T3–T4, high Ki-67).	Endocrine therapy ± ribociclib.	Broader eligibility; significant iDFS benefit.	[53]

## Data Availability

No new data were created or analyzed in this study. Data sharing is not applicable.

## Abbreviations

ALND	Axillary Lymph Node Dissection
ANC	Axillary node clearance
ART	Axillary radiotherapy
ASCO	American Society of Clinical Oncology
BC	Breast cancer
BCS	Breast-Conserving Surgery
BRCA	Breast Cancer Gene (BRCA1/BRCA2)
CDK4/6i	Cyclin-Dependent Kinase 4 and 6 Inhibitors
cN0	Clinically Node-Negative
cN+	Clinically Node-Positive
cT	Clinical tumor stage
ctDNA	Circulating Tumor DNA
DFS	Disease-Free Survival
DRFS	Distant relapse free survival
EBCTCG	Early Breast Cancer Trialists’ Collaborative Group
ER	Estrogen receptor
ESMO	European Society for Medical Oncology
ESR1	Estrogen Receptor 1
ET	Endocrine therapy
FNA	Fine Needle Aspiration
GII, GIII	Histological Grade II or III
gBRCA	Germline BRCA Mutation
HER2	Human Epidermal Growth Factor Receptor 2
HR+	Hormone Receptor-Positive
HRR	Homologous Recombination Repair
HT	Hormone Therapy
IBC	Inflammatory Breast Cancer
iDFS	Invasive disease-free survival
Ki67	Ki-67 Proliferation Index
LR	Local recurrence
LRFS	Local recurrence-free survival
MRI	Magnetic Resonance Imaging
mTOR	Mechanistic target of rapamycin
MSKCC	Memorial Sloan Kettering Cancer Center
NACT	Neoadjuvant Chemotherapy
NSAI	Non-steroidal aromatase inhibitor
NCCN	National Comprehensive Cancer Network
OS	Overall Survival
PARPi	Poly (ADP-ribose) Polymerase Inhibitors
PET/CT	Positron Emission Tomography/Computed Tomography
pN0	Pathologically Node-Negative
pN1	Pathologically Node-Positive (micrometastases or ≤2 positive nodes)
PIK3CA	Phosphatidylinositol-4,5-bisphosphate 3-kinase catalytic subunit alpha gene
PR	Progesterone receptor
RC	Capsular Rupture
RNI	Regional Nodal Irradiation
RS	Recurrence Score
RT	Radiation Therapy
SLNB	Sentinel Lymph Node Biopsy
T2, T3, T4	Tumor size classification (AJCC TNM staging)
TAS	Tailored Axillary Surgery
ycN0	Clinically node-negative after neoadjuvant therapy
